# Supplementary material for: Content-rich biological network constructed by mining PubMed abstracts
Source: BMC Bioinformatics. 2004 Oct 8;5:147. doi: 10.1186/1471-2105-5-147 (PMC528731; doi:10.1186/1471-2105-5-147)
Supplement: Additional File 2 — The original results of the above study (non-essential files are deleted to keep the file size under the limit set by BMC bioinformatics). [file 1471-2105-5-147-S2.bz2 › chilibotAdditionalFile2/dip10/439ID9507024E10388/html/CCL21_CCR7.html]

 


 **CCL21** and **CCR7** 
  
Found 97 abstracts in PubMed, retrieved 10.  
 

 What does Google say? 
 PDF only 
| .edu only 

---

**Interactive relationship** (e.g. stimulation, inhibition, etc)

**Neutral relationship**- In antigen trafficking from the skin, it has been postulated that Langerhans cells dendritic cells are activated after capturing exogenous antigens, up regulate the expression of the chemokine receptor,  **CCR7** , and migrate into lymphoid organs in response to the signaling of a chemokine,  **CCL21** , which is expressed in lymphatic vessels and T cell zone stromal cells.  Ref: 12750361 Int Immunol< MedlineTA>Int Immunol,
- These results identify endothelial  **CCL21**  expression as an important determinant for naive T cell migration to tertiary tissues, and suggest the  **CCL21**   **CCR7**  pathway as a therapeutic target in diseases that are associated with naive T cell recruitment.  Ref: 12707342 J Immunol, 2003

**Non-interactive relationship** (e.g. studied together, co-existance, homology, etc.)

- The CC chemokine receptor CCR 7  [ **CCR7** ]  ligands  **CCL21**  and CCL19 were recently described as essential elements for establishing the microenvironment needed to initiate optimal immune responses in secondary lymphoid tissues.  Ref: 12890630 Virology, 2003
- The binding to IP 10 could be inhibited by SLC  [ **CCL21** ]  but not by a non mac25 AGM binding chemokine, EBI1  [ **CCR7** ]  ligand chemokine ELC.  Ref: 12847218 J Immunol, 2003
- We show that the  **CCR7**  ligand CC chemokine ligand CCL 21  [ **CCL21** ]  is sufficient for attracting naive T cells into tertiary organs.  Ref: 12707342 J Immunol, 2003
- Here we demonstrate that there is a distinct pathway of antigen trafficking from skin in the steady state that is independent of  **CCL21**   **CCR7**  signaling.  Ref: 12750361 Int Immunol< MedlineTA>Int Immunol, 2003
- The increase in intracellular calcium in response to 6Ckine  [ **CCL21** ]  showed that chemokine receptor 7  [ **CCR7** ]  expression was functional.  Ref: 12682236 J Immunol, 2003
